# Darcy-Forchheimer Flow of Water Conveying Multi-Walled Carbon Nanoparticles through a Vertical Cleveland Z-Staggered Cavity Subject to Entropy Generation

**DOI:** 10.3390/mi13050744

**Published:** 2022-05-08

**Authors:** Ghulam Rasool, Abdulkafi Mohammed Saeed, Animasaun Isaac Lare, Aissa Abderrahmane, Kamel Guedri, Hanumesh Vaidya, Riadh Marzouki

**Affiliations:** 1 College of International Students, Wuxi University, Wuxi 214105, China; grasool@zju.edu.cn; 2 Institute of Intelligent Machinery, Faculty of Materials and Manufacturing, Beijing University of Technology, Beijing 100124, China; 3 Department of Mathematics, College of Science, Qassim University, Buraydah 51452, Saudi Arabia; abdulkafi.ahmed@qu.edu.sa; 4 Fluid Dynamics and Survey Research Group, Department of Mathematical Sciences, Federal University of Technology, Akure PMB 704, Nigeria; anizakph2007@gmail.com; 5 Laboratoire de Physique Quantique de la Matière et Modélisation Mathématique (LPQ3M), University of Mascara, Mascara 29000, Algeria; 6 Mechanical Engineering Department, College of Engineering and Islamic Architecture, Umm Al-Qura University, P.O. Box 5555, Makkah 21955, Saudi Arabia; kmguedri@uqu.edu.sa; 7 Department of Mathematics, Vijayanagara Sri Krishnadevaraya University, Ballari 583105, Karnataka, India; hanumeshvaidya@gmail.com; 8 Department of Chemistry, Faculty of Science, King Khalid University, P.O. Box 9004, Abha 61413, Saudi Arabia; rmarzouki@kku.edu.sa

**Keywords:** Darcy-Forchheimer flow, MWCNT-water nanofluid, vertical Cleveland Z-staggered cavity, entropy generation

## Abstract

To date, when considering the dynamics of water conveying multi-walled carbon nanoparticles (MWCNT) through a vertical Cleveland Z-staggered cavity where entropy generation plays a significant role, nothing is known about the increasing Reynold number, Hartmann number, and Darcy number when constant conduction occurs at both sides, but at different temperatures. The system-governing equations were solved using suitable models and the Galerkin Finite Element Method (GFEM). Based on the outcome of the simulation, it is worth noting that increasing the Reynold number causes the inertial force to be enhanced. The velocity of incompressible Darcy-Forchheimer flow at the middle vertical Cleveland Z-staggered cavity declines with a higher Reynold number. Enhancement in the Hartman number causes the velocity at the center of the vertical Cleveland Z-staggered cavity to be reduced due to the associated Lorentz force, which is absent when H_a_ = 0 and highly significant when H_a_ = 30. As the Reynold number grows, the Bejan number declines at various levels of the Hartmann number, but increases at multiple levels of the Darcy number.

## 1. Background Information

Nanofluids are fluids containing nano-sized particles in the base fluid-like substance such as oils, polymer solutions, biofluids, water, and lubricants. Nanofluids have recently been exposed by Choi and Eastman [1], Slimani et al. [2], Medebber et al. [3], Bendrer et al. [4], Zadeh et al. [5], and Aissa et al. [6] as a liquid substance capable to revolutionary heat transfer in several technical and industrial applications, such as domestic freezers, fuel cells, industrial mechanical processes, energy storage systems, and atomic reactors. Recently, Sahmeh et al. [7], Hussain et al. [8], and Muhammad et al. [9] used aqueous solutions to study various types of nanoparticles. Tlili et al. [10] have recently developed hybrid nanofluids and remarked on the higher heat transfer rates as compared to nanofluids. A hybrid nanofluid is a suspension of two types of nanoparticles in a base fluid. Microelectronics, propulsion, heat exchangers, military manufacturing, grinding, solar water heating, acoustics, transportation, and naval structures are all examples of hybrid nanofluid uses. Thanaa Elnaqeeb et al. [11] demonstrated a colloidal mixing of three different nanoparticles in ternary hybrid nanofluids. The viscosity of the five ternary hybrid nanofluid variants increases as the concentration rises. It was reported that when suction is small, the temperature distribution across ternary hybrid nanofluids is at a maximum. Heat transmission across the ternary-hybrid nanofluids with the density of a small nanoparticle is minimal because it is connected with greater levels of convective heating of ternary-hybrid nanofluids and generates a more significant heat transfer rate; an increase in the rate of convectively heating the wall is a factor capable of raising the temperature distribution; see Animasaun et al. [12].

Scrutinization of fluid dynamics in various cavities has recently been an impactful topic among scientists and technologists due to the enhancement of thermal performance in different thermal types of equipment. For instance, understanding the significance of fin attachment and the introduction of nanoparticles are two ways of controlling the heat transfer rate. Although, the research methodology for solving the coupled nonlinear governing equation is a severe challenge. Mansour et al. [13] realized that the heat transmission rate increased when the aspect ratio of a C-shaped cavity filled with nanofluids was reduced. In a study on Casson fluid flow due to double-diffusive natural convection in staggered cavities, Hussain et al. [14] discovered that when entropy creation is significant, the overall entropy output increases with increasing the Casson number. Zhang et al. [15] explored a cavity with a high-temperature L-shaped source using Fortran code based on the control volume approach and a simple algorithm. The smallest value of the Bejan number was found at a magnetic field of 15°. A change in the magnetic field can cause a change in Nusselt number of up to 53% and a change in produced entropy of up to 34%. Hosseinzadeh et al. [16] investigated heat transmission optimization in a hybrid nanofluid composed of MoS_2_–TiO_2_ nanoparticles in the center of an octagon with an elliptical cavity. The convective flow and the average Nusselt number fall as the Hartman number increases due to Lorentz forces and electrical vortices that resist fluid flow. The heat transfer rate in this environment deteriorates. Entropy analysis of Williamson nanofluid, unsteady nanofluid, and electrothermal couple stress nanofluid flow have been explained explicitly by Mandal and Shit [17,18,19]. Furthermore, increasing the parameter for quantifying the levels of thermal radiation from 0 to 0.8 reduces the average Nusselt number by 4.2%.

Sowmya et al. [20] investigated the transport of iron(ii) oxide and silver nanoparticles through water in a rectangular box with two heated fins on the bottom wall, where buoyancy and Lorentz forces are essential. Furthermore, when the Rayleigh number increases, so does the intensity of the velocity profile and streamlined function. Basak et al. [21] evaluated lid-driven mixed convection in a square chamber with four different heating temperatures. A GFEM with a penalty factor was used to simulate the nonlinear governing equations in the investigation. It is noteworthy that cooled walls, linear heating, and uniform heating affect average and local Nusselt numbers at various divisions. Furthermore, heat transmission was demonstrated to vary according to Darcy and Prandtl numbers (Da and Pr), whereas Reynolds numbers (Re) ranged between 10 and 102. The Darcy–Forchheimer–Brinkman confined domain boundary value problems are theoretical models for the flow of viscous incompressible fluids in porous cavities. Numerous issues of this type have been studied throughout history, but most notably in recent years, each with its technique, system, boundary condition, domain, and function space. However, the Darcy–Forchheimer flow of an mwcnt-water nanofluid in a vertical Cleveland Z-staggered cavity subject to entropy generation and continuous conduction on both sides has yet to be investigated. It is essential to know how the velocity, temperature, and entropy generation vary at different levels of Reynold number at different temperatures. What is/are the cause(s) of enhancement in the Hartman number on the velocity at the center of the vertical Cleveland Z-staggered cavity? In fact, due to an increasing Reynold number, it is necessary to determine the nature of variation in the: (a) Bejan number at different levels of Hartmann number; (b) maximum stream function value at different levels of Hartmann number; (c) Nusselt number at different levels of Hartmann number; (d) Nusselt number at different levels of Darcy number; (e) Bejan number at different levels of Darcy number; and (f) maximum stream function value at different levels of Darcy number. 

## 2. Research Methodology: Mathematical Formulation

**Figure 1:**
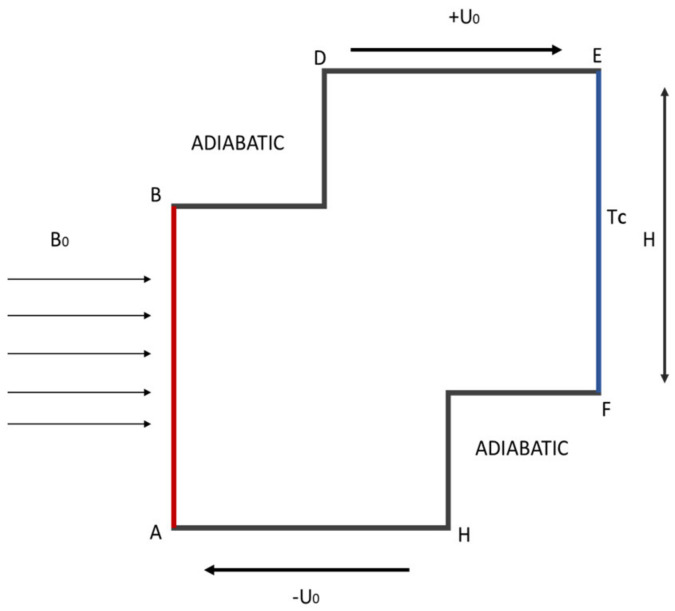
Illustration of 2D Darcy-Forchheimer flow through a vertical Cleveland Z-staggered cavity.

A two-dimensional staggered cavity with a length of L and width of W, as shown in Figure 1, where L and H are of equal size, was considered as the starting step for the research study. It was assumed that L_1_ and H_1_ are on an equal footing. At an angle of 90°, a B-intensity magnetic field was applied to the vertical Cleveland Z-staggered cavity. Constant conduction (Cc) and low temperature (Tc) was considered to impact a portion of the right wall of the length of H. Meanwhile, constant conduction (Ch) and the hot temperature were considered to influence the left wall (length of H) (Th). The top and lower walls of the vertical Cleveland Z-staggered cavity, as well as the rest of these walls, are adiabatic in nature.

### 2.1. Formulation of the Governing Equation

With the aim of providing answers to the research questions mentioned above, the Darcy–Forchheimer flow of water conveying multi-walled carbon nanoparticles through a vertical Cleveland Z-staggered cavity due to the ratio of buoyancy forces to flow shear forces was examined. Multi-walled carbon nanoparticles were considered due to their elongated cylindrical shape made of sp^2^ carbon; see Kukovecz et al. [22] for synthesis methods, as well as chemical and physical properties of multi-walled carbon nanotubes. The transport phenomenon is described as 2D, uniform, steady-state, incompressible, and laminar flow, where the cavity’s aspect ratio is AR = L1/L. The porous media is considered as homogenous and isotropic. Based on the preceding assumptions, the dimensionless forms of the *X*-momentum, *Y*-momentum, and the energy equation are:

(1) $$\frac{\partial U}{\partial X}+\frac{\partial V}{\partial V}=0,$$

(2) $$\frac{1}{\varepsilon^{2}}U\frac{\partial U}{\partial X}+\frac{1}{\varepsilon^{2}}V\frac{\partial U}{\partial Y}=-\frac{\partial P}{\partial X}+\frac{1}{R_{e}\cdot \varepsilon }\left[\frac{\partial^{2}U}{\partial X^{2}}+\frac{\partial^{2}U}{\partial Y^{2}}\right]-\frac{1}{R_{e}D_{a}}U-\frac{1.75}{\sqrt{150\varepsilon^{3}D_{a}}}U\sqrt{U^{2}+V^{2}},$$

(3) $$\begin{array}{c} \frac{1}{\varepsilon^{2}}U\frac{\partial V}{\partial X}+\frac{1}{\varepsilon^{2}}V\frac{\partial V}{\partial Y}=-\frac{\partial P}{\partial Y}+\frac{1}{R_{e}\cdot \varepsilon }\left[\frac{\partial^{2}V}{\partial X^{2}}+\frac{\partial^{2}V}{\partial Y^{2}}\right]-\frac{1}{R_{e}D_{a}}V-\\ \frac{1.75}{\sqrt{150\varepsilon^{3}D_{a}}}V\sqrt{U^{2}+V^{2}}+Ri\theta -\frac{\sigma_{nf}}{\sigma_{f}}\frac{\rho_{f}}{\rho_{nf}}\frac{Ha^{2}}{R_{e}}V, \end{array}$$

(4) $$U\frac{\partial \theta }{\partial X}+V\frac{\partial \theta }{\partial Y}=\frac{1}{R_{e}\cdot P_{r}}\left[\frac{\partial^{2}\theta }{\partial X^{2}}+\frac{\partial^{2}\theta }{\partial Y^{2}}\right]+\frac{Ra_{f}}{Ra_{E}R_{e}\cdot P_{r}}.$$

The equations mentioned above are produced by introducing the dimensionless variables listed below:

(5) $$X=\frac{x}{H},\;Y=\frac{y}{H},\;U=\frac{u}{H},\;V=\frac{v}{H},\theta =\frac{T-T_{c}}{T_{h}-T_{c}},\;\;\;\;P=\frac{p}{\rho U_{0}^{2}},\;\;\;\;\alpha =\frac{k_{x}}{\rho C_{p}}.$$

The emerged dimensionless parameters (i.e., Darcy number $D_{a}$, Prandtl number $P_{r}$, Reynold number $R_{e}$, Rayleigh number associated with the staggered fluid in cavity $Ra_{f}$, Rayleigh number associated with the entropy generation $Ra_{E}$, and Bejan number $B_{e}$) are defined as:

(6) $$\begin{array}{c} D_{a}=\frac{K}{H^{2}},\;P_{r}=\frac{v}{\alpha },\;R_{e}=\frac{U_{0}H}{v},\;Ra_{f}=\frac{g\beta q^{m^{\prime }}H^{5}}{v\alpha k_{x}},\;\mathrm{Ha}=\mathrm{LB}\sqrt{\frac{\sigma_{nf}}{\mu_{nf}}}.Ra_{E}=\frac{g\beta \left(T_{h}-T_{c}\right)H^{3}}{v\alpha },\\ B_{e}=\frac{\int S_{HT}dXdY}{\int S_{T}dXdY}=\frac{S_{HT}}{S_{T}},\;G_{r}=\frac{g\beta H^{3}\left(T_{k}-T_{c}\right)}{v^{2}},\;Ri=\frac{G_{r}}{R_{e}R_{e}} \end{array}$$

The thermo-physical characteristics of the water conveying multi-walled carbon nanoparticles presented in Table 1 are defined as:

(7) $$\begin{array}{c} \rho_{\mathrm{hnf}\;}=\left(1-\phi \right)\rho_{f}+\phi \rho_{p},\;{\left(\rho \beta \right)}_{\mathrm{hnf}\;}=\left(1-\phi \right){\left(\rho \beta \right)}_{f}+\phi {\left(\rho \beta \right)}_{p},\;\mu_{\mathrm{hnf}}{\left(1-\varphi \right)}^{2.5}=\mu_{bf}\\ {\left(\rho C_{p}\right)}_{\mathrm{hnf}\;}=\left(1-\phi \right){\left(\rho C_{p}\right)}_{f}+\phi {\left(\rho C_{p}\right)}_{p},\;\frac{k_{\mathrm{hnf}\;}}{k_{f}}=\frac{k_{np}+\left(n-1\right)k_{f}-\left(n-1\right)\left(k_{f}-k_{np}\right)\varphi }{k_{np}+\left(n-1\right)k_{f}+\left(k_{f}-k_{np}\right)\varphi }\;,\\ \alpha_{\mathrm{hnf}\;}=\frac{k_{\mathrm{hnf}\;}}{{\left(\rho C_{p}\right)}_{\mathrm{hnf}\;}}. \end{array}$$

The dimensionless boundary conditions associated with Equations (1)–(4) for the hot wall:

(8) U=0,  V=0,  θ=1,

for the cold wall:

(9) U=0,  V=0,  θ=0,

for the moving wall:

(10) $$\frac{\partial \theta }{\partial Y}=0,\;\;U=1,\;\;V=0,$$

for the stationary adiabatic walls:

(11) $$\frac{\partial \theta }{\partial Y}=0,\;\;U=0,\;\;V=0,$$

The mean and local Nusselt numbers are of the form:

(12) $$Nu_{m}=\int_{0}^{1}NudY\;\;\;\;\;\;\;Nu=-\frac{\partial \theta }{\partial X}.$$

The expression in the model to account for the amount of entropy that was produced during the irreversible process associated with the dynamics in the staggered cavity is:

(13) $$\begin{array}{c} S_{T}=\frac{k_{nf}}{k_{f}}\left[{\left(\frac{\partial \theta }{\partial X}\right)}^{2}+{\left(\frac{\partial \theta }{\partial Y}\right)}^{2}\right]+\\ \frac{\mu_{nf}}{\mu_{f}}x\left\{2\left[{\left(\frac{\partial U}{\partial X}\right)}^{2}+2{\left(\frac{\partial V}{\partial Y}\right)}^{2}\right]+{\left(\frac{\partial U}{\partial Y}+\frac{\partial V}{\partial X}\right)}^{2}+\chi {\mathrm{Ha}}^{2}\frac{\sigma_{nf}}{\sigma_{f}}\left(U^{2}+V^{2}\right)\right\} \end{array}$$

whereas,

(14) $$x=\frac{\mu_{f}T_{0}}{k_{f}}{\left(\frac{u_{w}}{T_{h}-T_{c}}\right)}^{2}$$

(15) $$S_{T}=S_{HT}+S_{FF}+S_{MF}$$

where the entropy production due to heat transfer irreversibility, *S_HT_*, magnetic field, *S_MF_*, and fluid friction irreversibility, *S_FF_*, are defined as:

(16) $$\begin{array}{c} S_{HT}=\frac{k_{nf}}{k_{f}}\left[{\left(\frac{\partial \theta }{\partial X}\right)}^{2}+{\left(\frac{\partial \theta }{\partial Y}\right)}^{2}\right],\;\;S_{FF}=\frac{\mu_{nf}}{\mu_{f}}\chi \left\{2\left[{\left(\frac{\partial U}{\partial X}\right)}^{2}+2{\left(\frac{\partial V}{\partial Y}\right)}^{2}\right]+{\left(\frac{\partial U}{\partial Y}+\frac{\partial V}{\partial X}\right)}^{2}\right\},\\ S_{MF}=\chi {\mathrm{Ha}}^{2}\frac{\sigma_{nf}}{\sigma_{f}}\left(U^{2}+V^{2}\right) \end{array}$$

### 2.2. Solution Methodology and Validation

**Figure 2:**
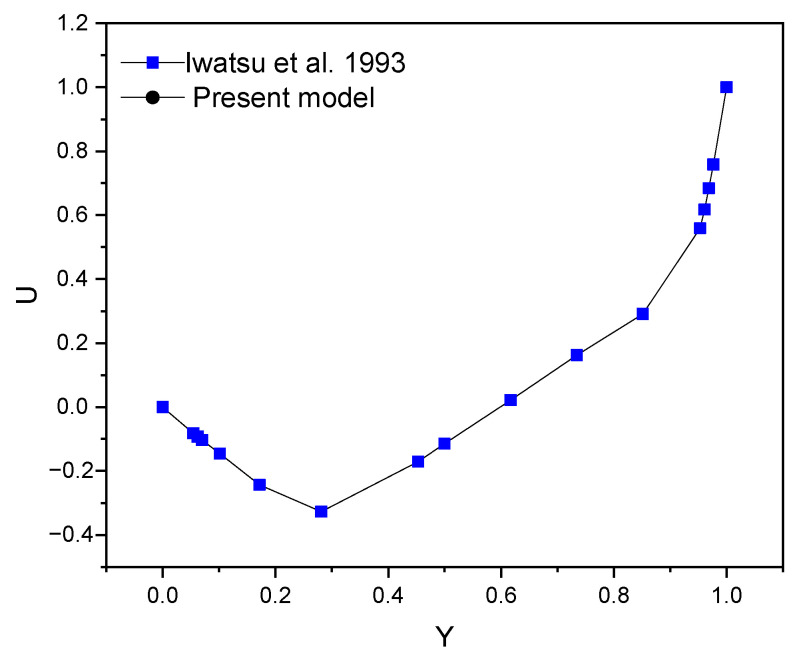
Validation of the code velocity profile (Comparison of a new result with that of Iwastu et al. [25] for a limiting case).

The solution starts with a basis function $M_{i}$ and its coefficients $N_{i}$ to be determined in

$$\hat{u}=\overset{n}{\underset{i=1}{\sum }}M_{i}N_{i}$$

such that:

$$\overset{}{\underset{V}{\int }}\phi \left(L\hat{u}-P\right)dV=0$$

foe every function

$$\phi =\overset{n}{\underset{i=1}{\sum }}\phi_{i}M_{i}$$

where $\phi_{i}$ are arbitrary coefficients and ϕ must satify the boundary conditions homogeneously. The resulting solutions of each equation $N_{i}$ yields the approximate solution of $\hat{u}$. The unknown functions that satisfy governing Equations (1)–(4) are subject to and associated with conditions Equations (8)–(11) and were obtained using the Galerkin-weighted residual finite element technique suggested by Bendrer et al. [23] and Al-Kouz et al. [24]. Numerous grids were evaluated. As shown in Table 2, the observed findings convinced us to employ an extra-fine grid with 22,414 triangular pieces in the present investigation. In order to ensure the numerical approach used in the code is accurate, the velocity profile is depicted and compared against the findings reported by Iwastu et al. [25], as seen in Figure 2.

## 3. Analysis and Discussion of Results

**Figure 3:**
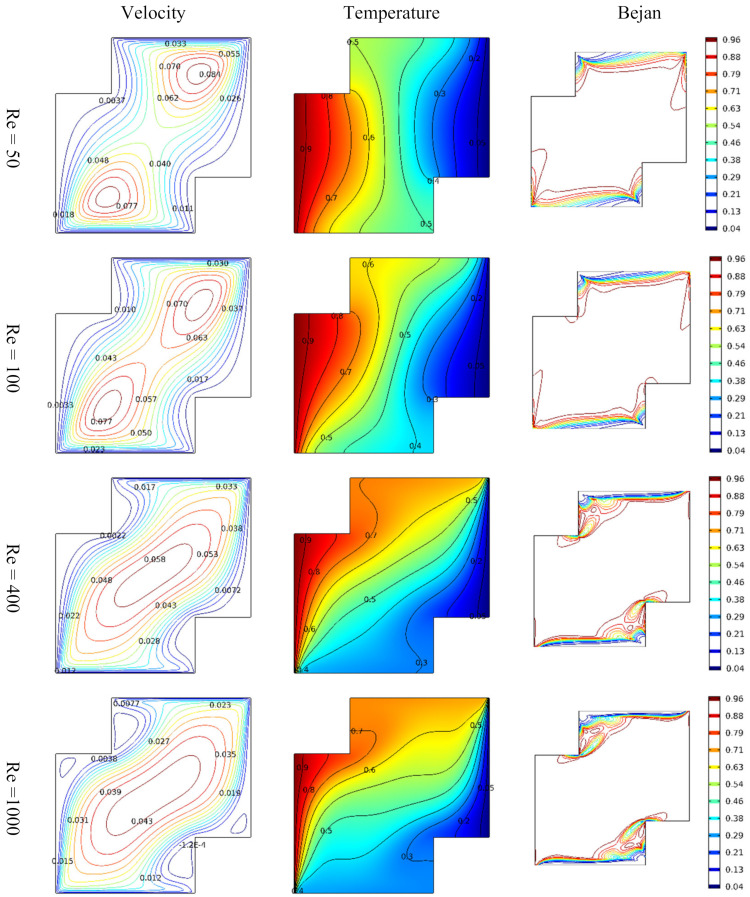
Variations in the velocity, temperature distribution, and entropy generation at different levels of Reynold number.

At different levels of Reynold number, as illustrated in Figure 3, minimum temperature occurs near the right-hand side of the vertical Cleveland Z-staggered cavity of low temperature. It is evident that increasing the magnitude of Reynold number causes the pattern of variation in the temperature at the middle to further slant away from the hot side to the cold side. It may be concluded that increasing the Reynold number causes the inertial force to be enhanced. Consequently, increasing the inertial force affects the distribution of heat energy from continuous conduction, but from the heated side to the cool side. The Reynolds number determines whether a fluid flow is laminar or turbulent. Due to the larger magnitude of inertia force, an object with a higher Reynolds number can force its way through a flow field. The result shows that an increase in *R_e_* less than 2000 corresponds to a larger inertia force; see Rapp [26]. The incompressible Darcy–Forchheimer flow of MWCNT-water nanofluid in a vertical Cleveland Z-staggered cavity is observed to be affected by increasing Reynold number as illustrated in Figure 3. For instance, the domain of maximum velocity occurs at just two points in the domain when *R_e_* = 50. As *R_e_* = 1000, the domain of maximum velocity enlarges at the middle of the vertical Cleveland Z-staggered cavity. However, it is worth noting that the magnitude of the maximum velocity ($v_{m}$) declined at the middle of the vertical Cleveland Z-staggered cavity as $R_{e}\to 1000$. Using the technique of slope linear regression through the data points announced in references [27,28,29] shows that the observed rate in a decrease in $v_{m}$ with $R_{e}$ is −3.74426 × 10^−5^. In compact form, $\left[R_{e},v_{m}\right]$ are $\left[50,\;0.084\right]$, $\left[100,\;0.070\right]$, $\left[400,\;0.058\right]$, and $\left[1000,\;0.043\right]$. 

**Figure 4:**
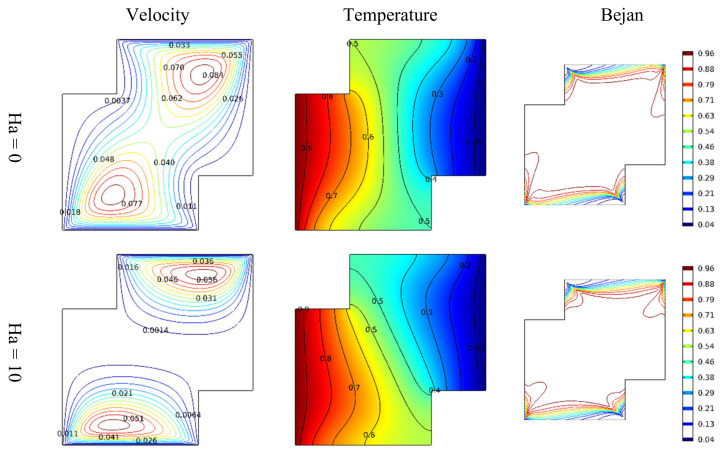

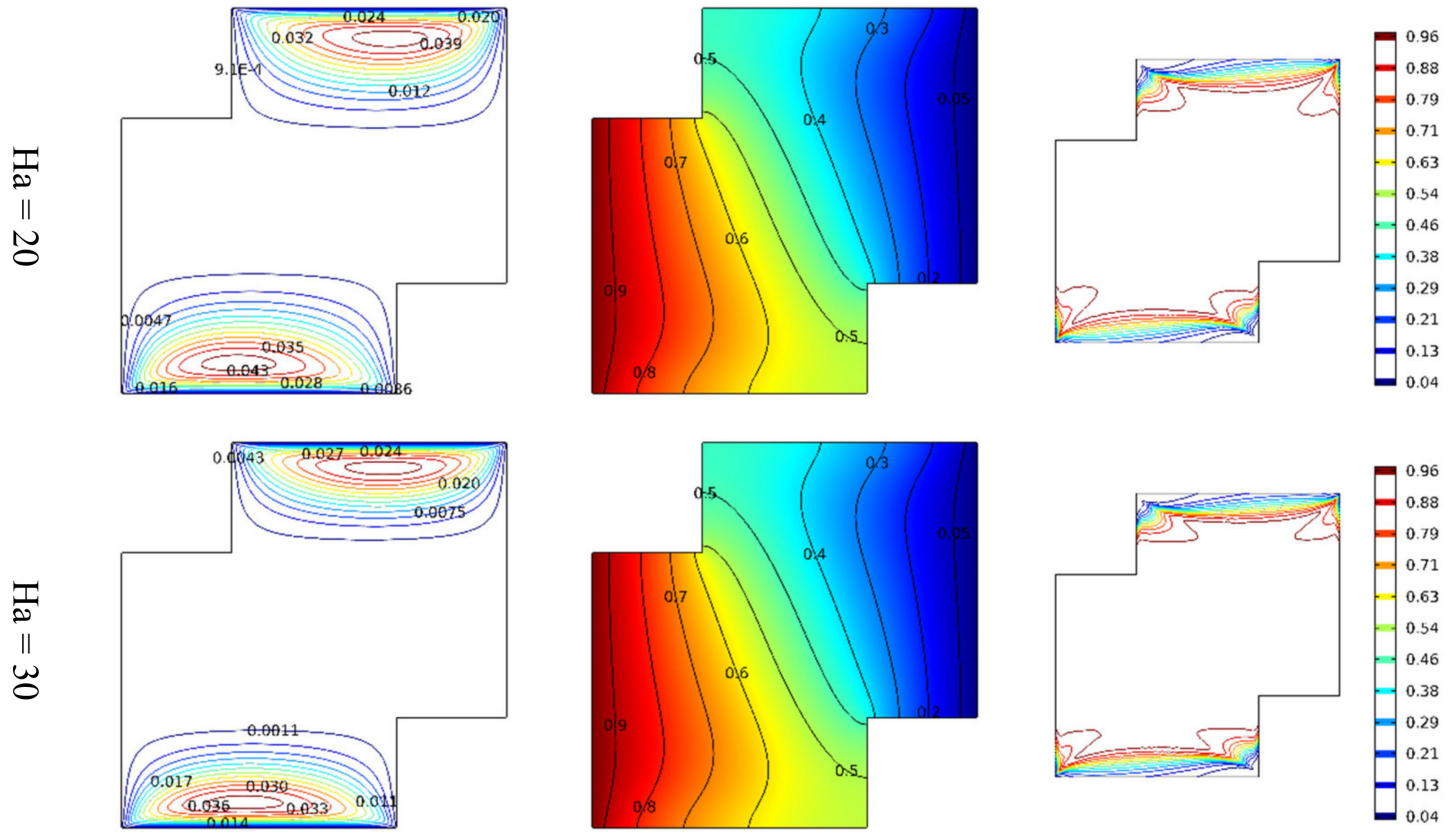
Variations in the velocity, temperature distribution, and entropy generation at different levels of Hartmann number.

**Figure 5:**
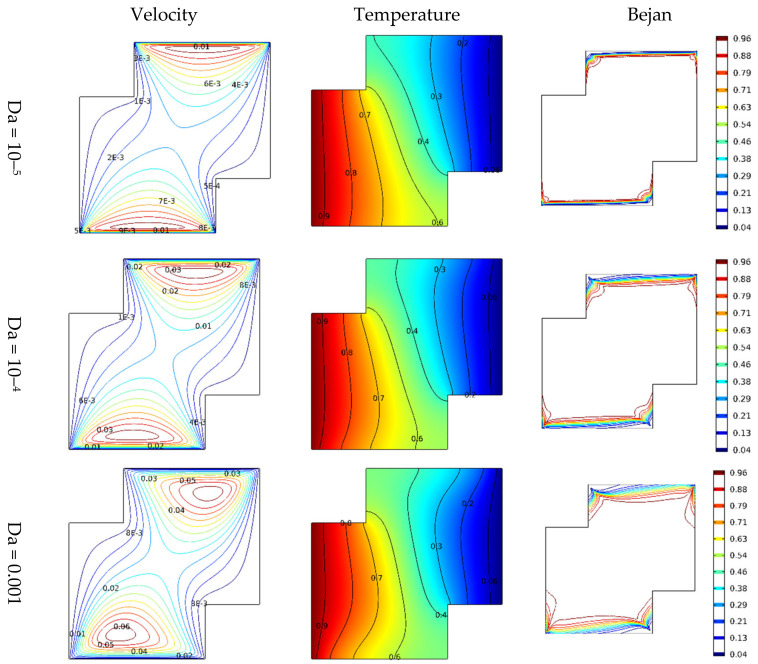

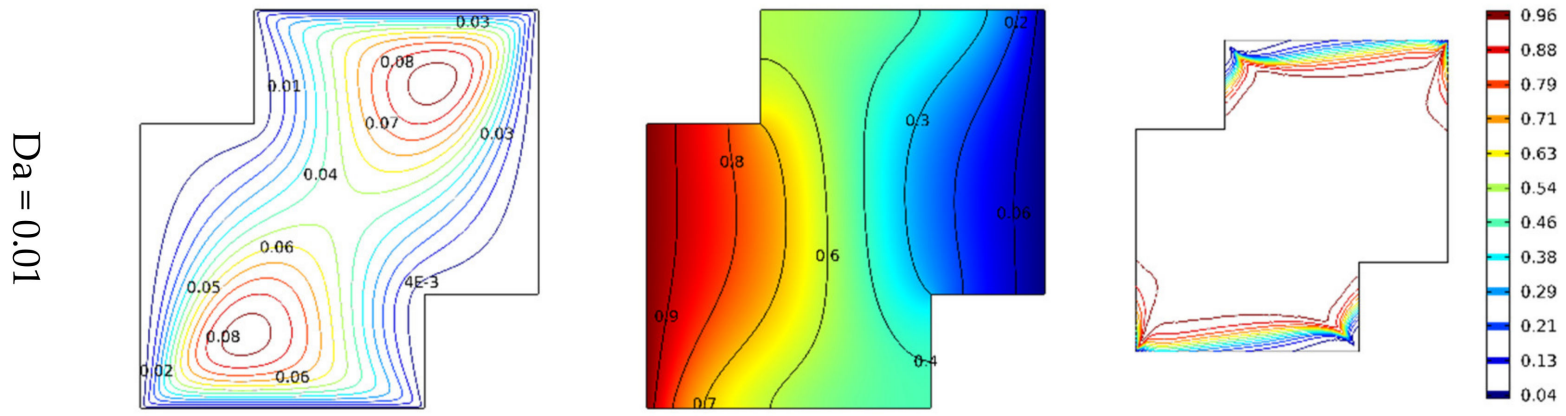
Variations in the velocity, temperature distribution, and entropy generation at different levels of Darcy number.

**Figure 6:**
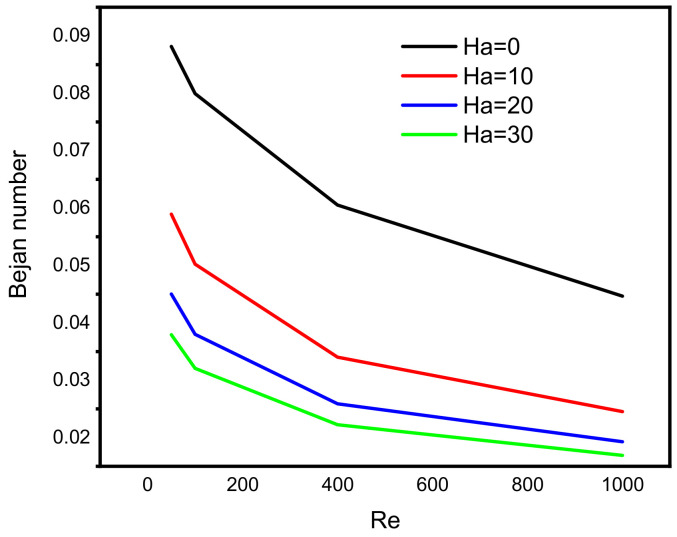
Variation in Bejan number with Reynold number at different levels of Hartmann number.

**Figure 7:**
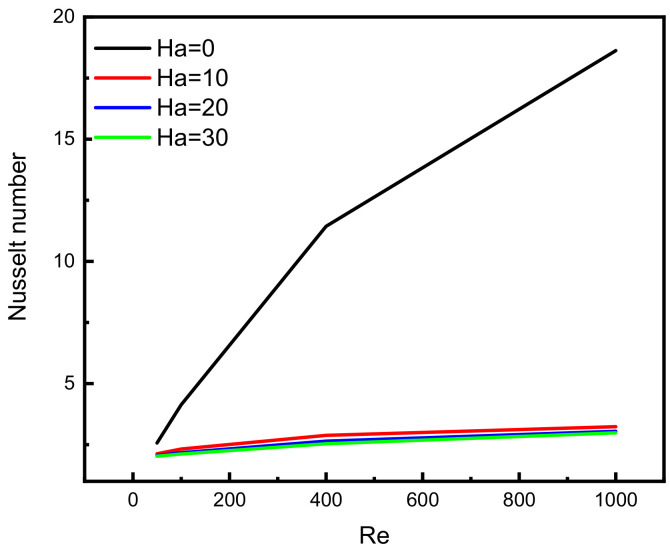
Variation in Nusselt number with Reynold number at different levels of Hartmann number.

The observation above suggests that the velocity of incompressible Darcy-Forchheimer flow of MWCNT-water nanofluid at the middle of a vertical Cleveland Z-staggered cavity declines with a higher Reynold number due to the enhancement of the associated inertial force. As the magnitude of Reynold number increases, Al kouz et al. [24] once discovered that the inadequacy of coarse meshes gradually becomes apparent. The results in Figure 4 show that the greater the Hartmann number, the more the velocity at the cavity’s center is slowed. For instance, in the absence of the associated Lorentz force (i.e., when Ha = 0), the outcome of this study shows the possibility of getting the velocities 0.062 and 0.040 at the middle of the vertical Cleveland Z-staggered cavity. As the magnitude of the Hartmann number increases (i.e., the higher the Lorentz force), the velocity at the middle reduces from 0.014 (when Ha = 10) to 0.012 (when Ha = 20), and 0.0011 when Ha = 30. Near the hot and cold wall, the distribution of heat energy is maintained. In a study on the magnetic field on the peristaltic transport of blood in a non-uniform setting, Mekheimer [30] noticed that the ratio of force to the area is an increasing property of the Hartmann number. Figure 5 reveals that the increasing Darcy number influences the velocity function. As expected, the temperature distribution is a constant function of Darcy number. In a study on the influence of increasing Darcy number, Marcelo [31] discovered that a larger interfacial heat transfer area boosts energy transfer throughout the channel, resulting in a more effective heat exchange between phases. This scientific fact supports the newly acquired results illustrated in the third column of Figure 5. At the upper and lower bounds of the vertical Cleveland Z-staggered cavity, the domain covered by the Bejan number enlarges as the magnitude of Darcy number increases. Figure 6 reveals that the Bejan number decreases with Reynold number. A higher decreasing trend in Bejan number with Reynold number manifests when Ha = 0 (absence of Lorentz force). It is also discovered, see Figure 7, that the Nusselt number that quantifies the heat transfer rate grows insignificantly with the Reynold number at all levels of increasing Hartmann number.

**Figure 8:**
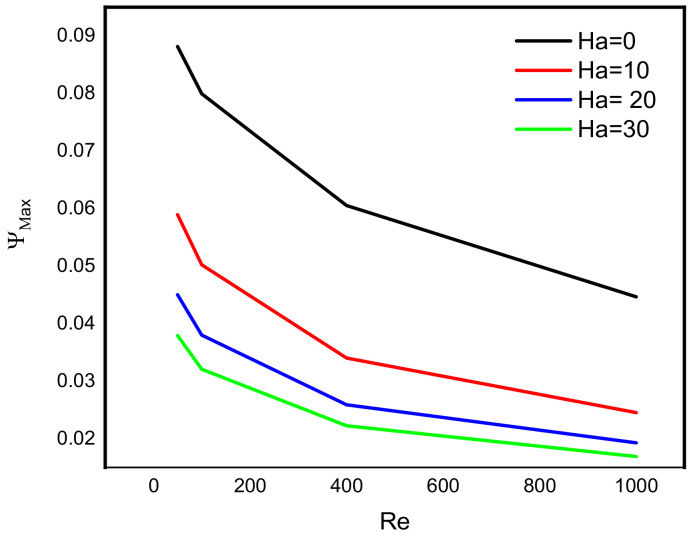
Variation in maximum stream function value with Reynold number at different levels of Hartmann number.

**Figure 9:**
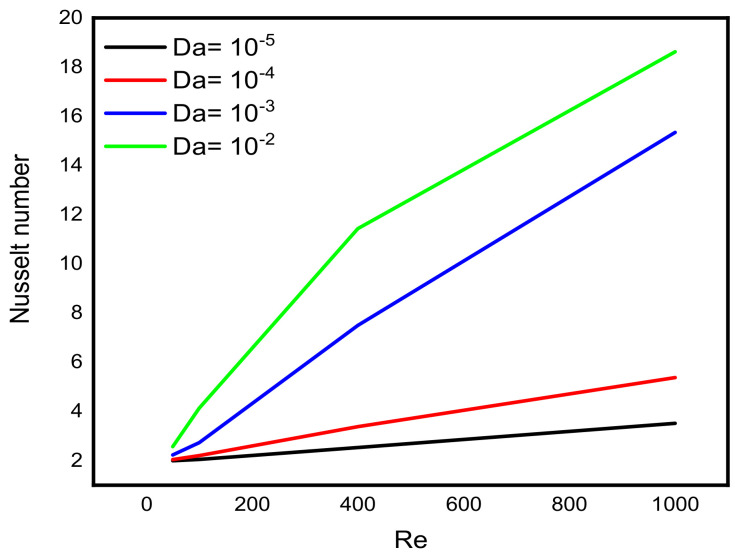
Variation in the Nusselt number with Reynold number at different levels of Darcy number.

**Figure 10:**
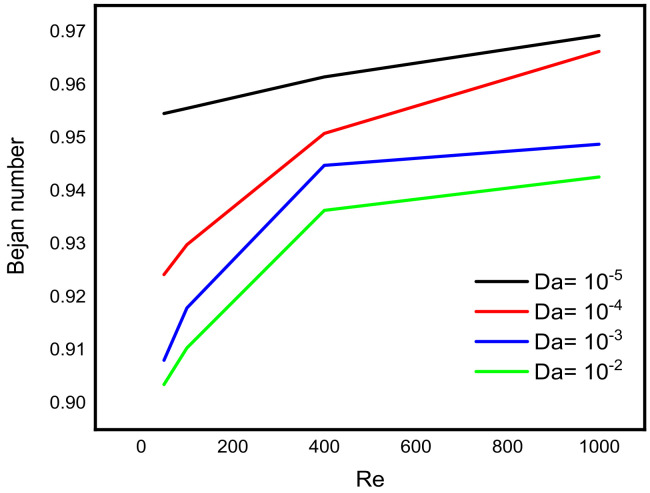
Variation in the Bejan number with Reynold number at different levels of Darcy number.

**Figure 11:**
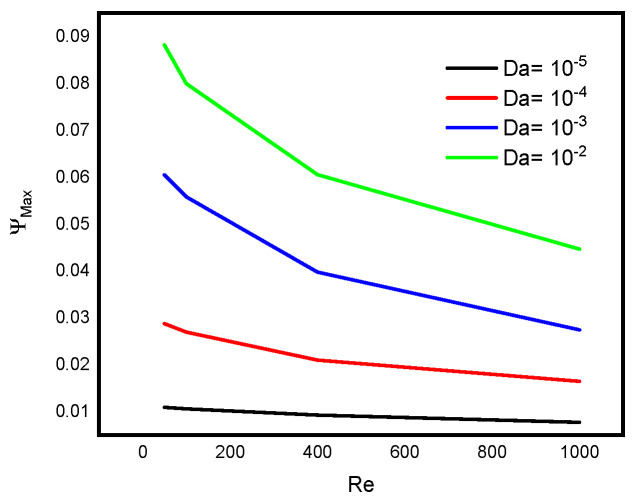
Variation in the maximum stream function value with Reynold number at different levels of Darcy number.

Intensive increasing of the Nusselt number at each level of the Reynold number is observable when the Lorentz force is infinitesimal. According to Sheikholeslami [32], as the Reynolds number rises, the isotherms near the lid wall would get denser due to convection enhancement. More specifically, as the Darcy number rises, the temperature gradient across the hot wall also rises. Figure 8 reveals a decreasing pattern of the maximum stream function value with Reynold number at different levels of Darcy number. Since the force exerted on a charged particle moving with velocity through a magnetic field and an electric field is perpendicular to the flow of Darcy-Forchheimer MWCNT-water through a vertical Cleveland Z-staggered cavity, the maximum stream function value decreases with increasing Reynold number; see Figure 8. It is worth noticing from Figure 9 that the Nusselt number was found to be an increasing function of Reynold number at each level of Darcy number. It is worthy to note that the rate of increase in the Nuselt number with Reynold number is minimal when Darcy number is minimal. The isotherms get less dense as the Lorentz forces increase. Furthermore, as the Hartmann number rises, velocity falls. According to Kandelousi and Ganji [33], high permeability promotes robust flow circulation in the enclosure, whereas low permeability inhibits flow circulation and results in a weak flow. Based on the stream function value, it is clear that lowering Da from 0.1 to 0.001 inhibits flow circulation within the domain and reduces the stream function for the fluid domain. Figure 10 and Figure 11 reveal that the Bejan number increases while the maximum stream function value decreases with Reynold number.

## 4. Conclusions

The dynamics of Darcy–Forchheimer flow of water conveying multi-walled carbon nanoparticles through a vertical Cleveland Z-staggered cavity subject to entropy generation and constant conduction on both sides, but at different temperature levels has been investigated. It is worthy to conclude that:

(a) Increasing the Reynold number causes the inertial force to be enhanced. Consequently, increasing the inertial force affects the distribution of heat energy from constant conduction, but from the heated side to the cool side;
(b) The velocity of incompressible Darcy–Forchheimer flow of water conveying multi-walled carbon nanoparticles at the middle of the vertical Cleveland Z-staggered cavity declines with a higher Reynold number due to enhancement of the associated inertial force;
(c) Enhancement in the Hartman number causes the velocity at the center of the vertical Cleveland Z-staggered cavity to be reduced due to the associated Lorentz force, which is absent when Ha = 0 and highly significant when Ha = 30;
(d) The higher the Darcy number, the greater the velocity function increase, but only at the middle of the vertical Cleveland Z-staggered cavity.
(e) As the Reynold number grows,
  - the Bejan number declines at various levels of Hartmann number, but increases at various levels of Darcy number;
  - the Nusselt number increases significantly at various levels of Darcy number, but negligibly at various levels of Hartmann number;
  - The maximum stream function value diminishes at various levels of Hartmann and Darcy numbers.

An extension of the study is to unravel the dynamics of ternary-hybrid nanofluid through a vertical Cleveland Z-staggered cavity and is recommended for a deeper understanding of the effects of entropy generation, Darcy number, and Reynold number.

## Figures and Tables

**Table 1:** Nanoparticles and base fluid thermo-physical properties as per Bendrer et al. [23].

	*Pure Water*	*Mwcnt*
ρ (kg/m^3^)	997.1	2100
*C_p_* (J/kg k)	4179	710
k (W/m k)	0.613	2000
σ (S/m)	5.5 × 10^−6^	1.9 × 10^−4^

**Table 2:** Comparison between Nu_avg_ for different grid resolutions.

	Number of Grids			
	3498	8508	22,414	27,916
*R_e_* = 500	3.3784	3.3811	3.3813	3.3817
*R_e_* = 1000	3.5195	3.5230	3.5236	3.5236

## Data Availability

Data would be made available upon a reasonable request.
